# Supplementary figures and images for: Integrating GWAS with bulk and single-cell RNA-sequencing reveals a role for LY86 in the anti-Candida host response
Source: PLoS Pathog. 2020 Apr 6;16(4):e1008408. doi: 10.1371/journal.ppat.1008408 (PMC7173933; doi:10.1371/journal.ppat.1008408)

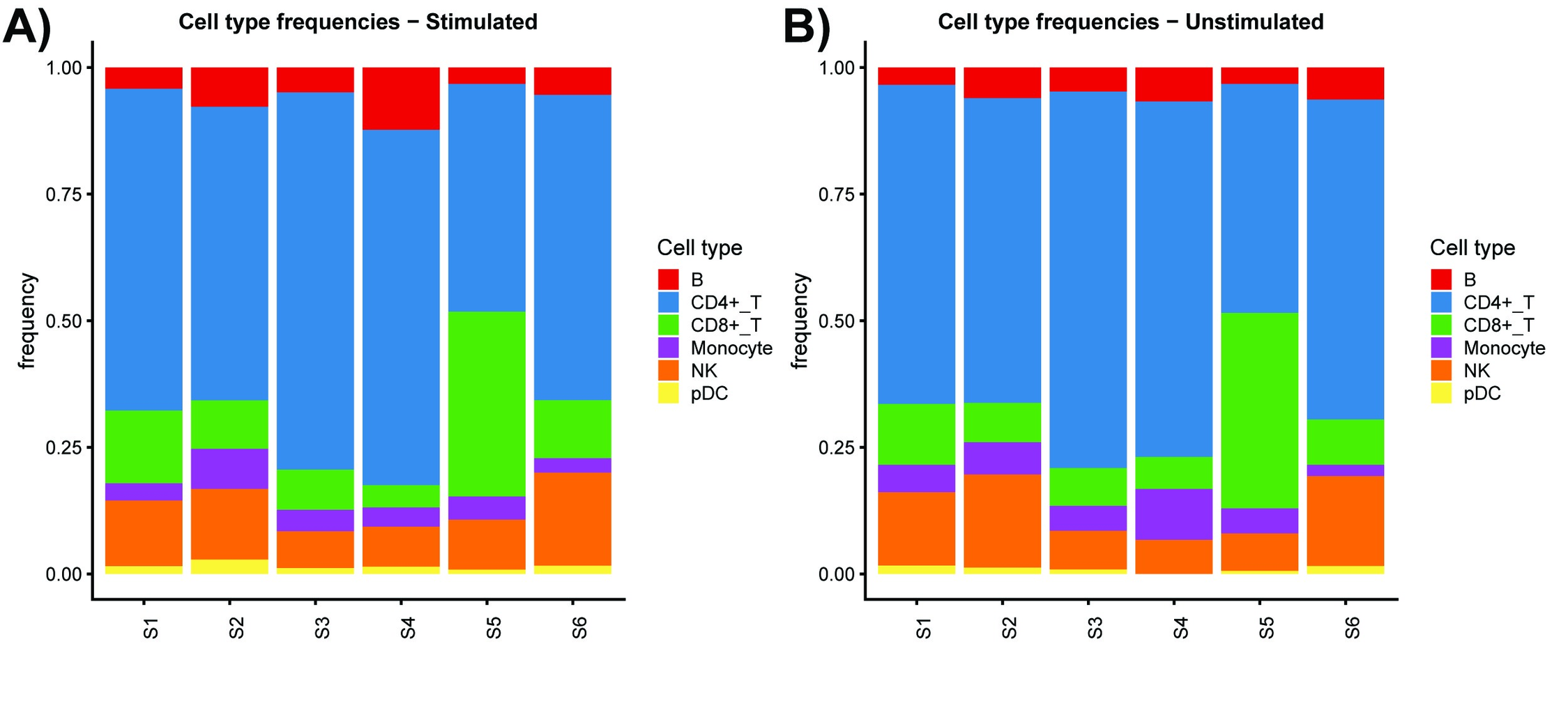

Supplement: S1 Fig — The relative cell type frequency per donor (A) before and (B) after 24h Candida stimulation for each of the 6 cell types identified within the peripheral blood mononuclear cells: B-cells, CD4+ and CD8+ T cells, monocytes, natural killer (NK) cells and plasmacytoid dendritic cells (pDCs). (TIF) [file ppat.1008408.s001.tif]

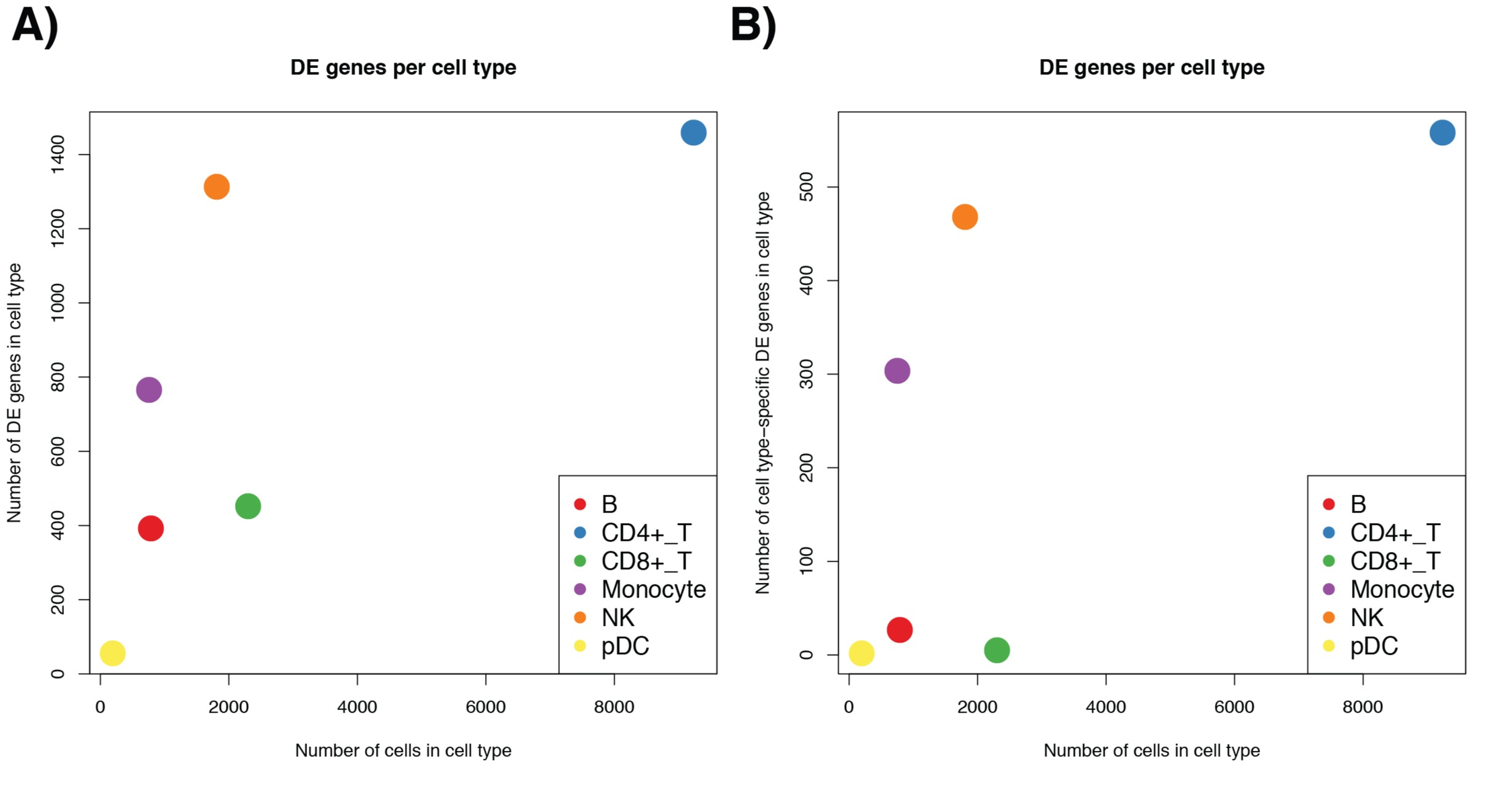

Supplement: S2 Fig — The number of differentially expressed (DE) genes per cell type (A) for all and (B) for the unique DE genes against the number of cells. (TIF) [file ppat.1008408.s002.tif]

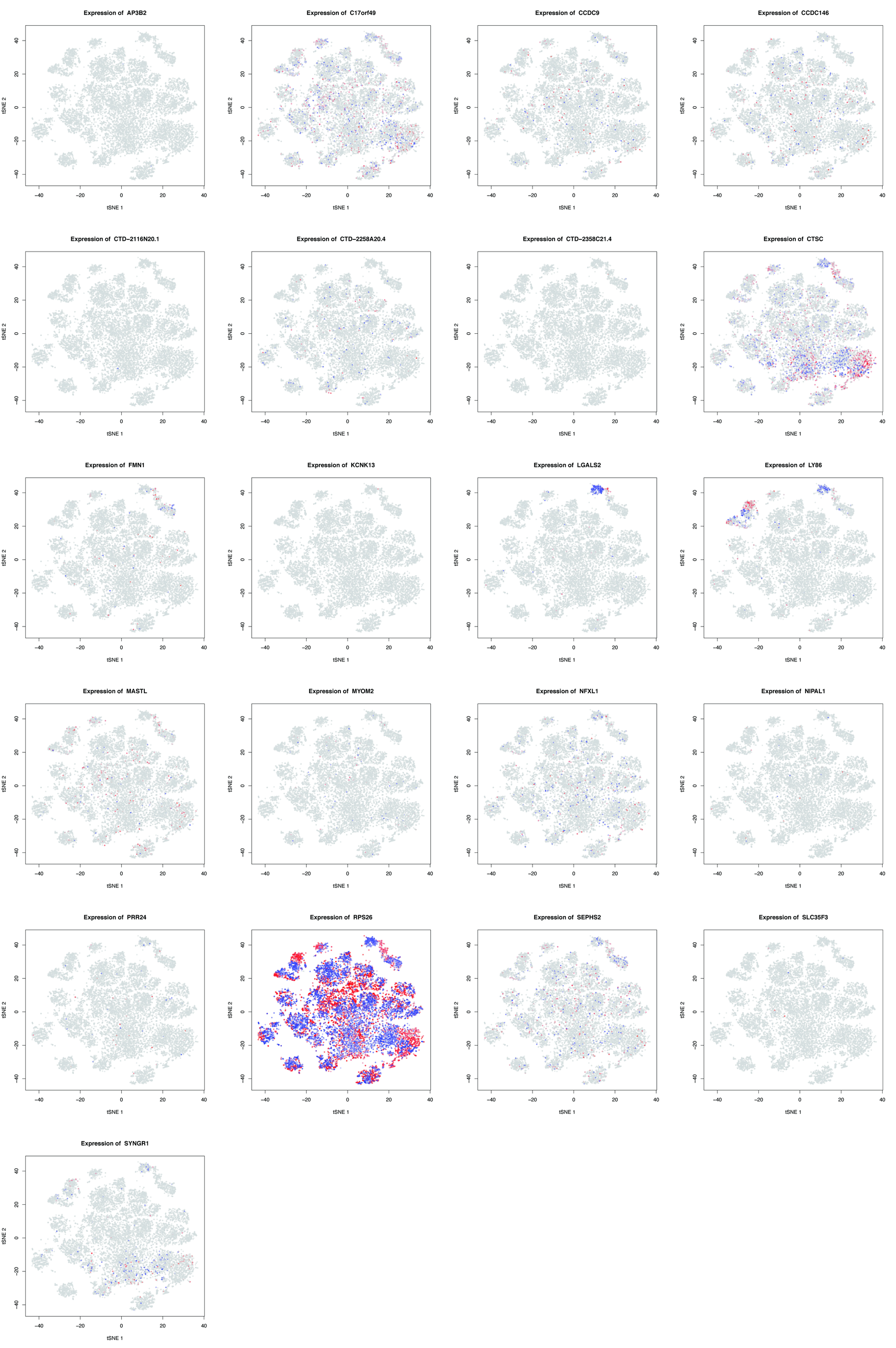

Supplement: S3 Fig — 27 response QTLs were identified in PBMC bulk RNA-seq data of 24h Candida-stimulated cells compared to RPMI control cells. To pinpoint the cell type in which the response QTL effect could manifest itself, PBMC single-cell RNA-seq data of 24h Candida-stimulated cells compared to RPMI control cells was used. For 21 out of the 27 response QTL genes, expression was detected in the single-cell RNA-seq data. The expression of individual cells was colored according to the condition: red for stimulated and blue for RPMI control cells. The expression level is colored by intensity, with gray cells having no expression. (TIF) [file ppat.1008408.s003.tif]

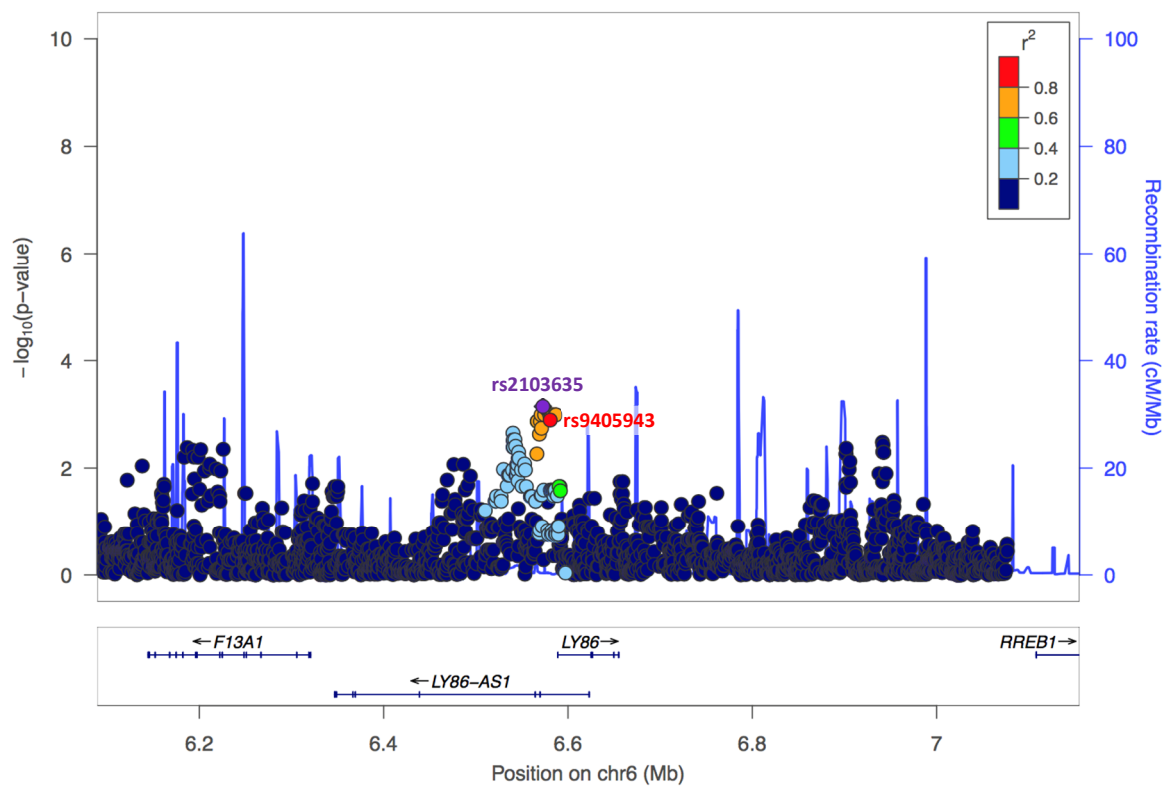

Supplement: S4 Fig — Regional association plot of the LY86 locus (chr6;6088933–7155216, build hg19, ± 500 kb). P values on the -log10 scale are presented as a function of the chromosomal position. The top enriched Candida-response QTL SNP rs9405943 is presented as the red circle and the SNP rs2103635 showing the strongest association to candidemia susceptibility is shown as the purple diamond. The correlations (r2) of each of the surrounding SNPs to SNP rs2103635 are shown in the indicated colors. Recombination rate is shown in pale blue. (PDF) [file ppat.1008408.s004.pdf]
