# Supplementary material for: Integrating GWAS with bulk and single-cell RNA-sequencing reveals a role for LY86 in the anti-Candida host response
Source: PLoS Pathog. 2020 Apr 6;16(4):e1008408. doi: 10.1371/journal.ppat.1008408 (PMC7173933; doi:10.1371/journal.ppat.1008408)
Supplement: Attachment — Submitted filename: RebuttalReviewerComments.docx [file ppat.1008408.s010.docx]

**Reply to reviewer comments**

We would like to thank the reviewers for their highly constructive comments and suggestions, which have enabled us to improve our manuscript substantially. We believe that we have now adequately addressed each of the comments. In short, we have now experimentally validated the proposed working mechanism of *LY86*. Additionally, through ligand-receptor expression analysis we have provided additional support for an important role of NK cells in the immune response against *Candida*. Finally, we have increased the clarity of the figures and their legends, and resolved any unclarities in the main text. We have provided a point-point reply to each of the questions that have been raised. Reviewer comments are in **Verdana Bold**, our responses are in plain Verdana, and adjustments to the text in *cursive* *Verdana*.

**Reviewer's Responses to Questions**

**Part I – Summary: Please use this section to discuss strengths/weaknesses of study, novelty/significance, general execution and scholarship.**

**Reviewer #1: This manuscript addresses an important problem, Candida bacteremia, by combining GWAS and RNA-seq. Their approach revealed 27 Candidemia response eQTLs and a plausible GWAS hit near LY86. For scRNA-seq, the investigators used PBMCs from 6 individuals and stimulated with Candida ex vivo. Bulk RNA-seq was from a previous published study (Oosting et al 2016 in Nature Med) and cis eQTLs from an unstimulated cohort of 31684 people was also from a previously published study, and the GWAS was also previously published.**

The bulk RNA-seq (Li et al., 2016) and GWAS study (Jaeger et al., 2019) have been published before elsewhere. However, we now used this bulk RNA-seq data to perform a *cis*-response-QTL analysis, whereas previously it was just used for DE analysis. To increase the power to detect response-QTLs in this relatively small dataset (~70 individuals), we made use of the top *cis*-eQTLs that were identified before in a cohort of 31,684 blood samples. Please note that this cohort of 31,684 samples contains unstimulated blood samples, whereas using the *Candida*-stimulated bulk RNA-seq study in ~70 individuals we can now determine whether these *cis*-eQTLs have a stronger response upon stimulation with *Candida* (i.e. are response-QTLs).

We discuss the above at p7-8, line 175-179: *‘The rather small sample size of this study limits its predictive power, in part by the large multiple testing burden of genome-wide eQTL analysis [22]. To reduce the multiple testing burden, we limited our single nucleotide polymorphism (SNP)-gene combinations to only the 16,990 top cis SNP-gene pairs identified in the largest eQTL meta-analysis to date [23], containing unstimulated whole blood samples of 31,684 individuals.’*

Additionally, since the GWAS study contains a fairly limited number of individuals (161 candidemia cases and 152 disease-matched controls), the study by itself does not have a lot of power to detect genome-wide significant SNPs associated with candidemia. As small patient group sizes are limiting our ability to gain a further increase in disease understanding, we used a different approach to further our understanding of candidemia. In this manuscript we show that through an integrative systems genetics approach, in which multiple layers of information are combined – that by themselves are not necessarily genome-wide significant – we can gain additional insights in the mechanisms underlying the immune response to *Candida* infection.

We highlight the above at p3, line 65-67: ‘*However, in the context of candidemia and other infectious diseases, conducting a GWAS is challenging due to the limited size of patient cohorts [8]*.’ And at p8, line 196-201: ‘*Previously, it was shown that integrating multiple molecular datasets can help prioritize disease-relevant genes, cell types and pathways [9,10]. Therefore, as a next step, we took the GWAS summary statistics of a previously published candidemia cohort of 161 cases and 152 disease-matched controls [8] and overlaid this with our 27 response QTLs (Fig. 2B). This revealed an enrichment of candidemia-susceptibility SNPs within the Candida-response QTL SNPs (ƛ_inflation_ = 1.49) (Fig. 2C).’*

**As expected, they observed thousands of differentially expressed genes, and they combined their transcriptomics data with a small published GWAS to develop a hypothesis regarding LY86 and host response to Candida.**

**I appreciate that the scRNA-seq data being generated are valuable, as is integrating this study with other published data, but the current state of this study does not lead to any substantial biological insight. Some interesting hypotheses, particularly regarding LY86 are generated, but these ideas undergo no testing. The method itself appears useful, though restricting search space to eQTLs, such as with stratified QQ plots is a fairly standard approach. Thus, I feel that the work has limited impact.**

In addition to creating the scRNA-seq data and its integration with other published data, we also performed a new response-QTL analysis on the bulk RNA-seq data from (Li et al., 2016), identifying 27 novel Candida response-QTLs. By integrating all the datasets and analyses we were able to provide additional insights in the involvement of individual blood cell types in the immune response against *Candida* (by overlapping response-QTLs with scRNA-seq data and by performing DE and receptor-ligand expression analysis in the scRNA-seq data). Moreover, in this revised manuscript we have now conducted experimental follow-up on the hypothesis that was being generated by our integrative approach (i.e. reduced expression of *LY86* upon stimulation with *Candida* *–* associated with having the candidemia-risk allele at SNP rs2103635 – reduces the migratory capacity of monocytes as a consequence of reduced expression of *CCR2*). These experiments confirmed our hypothesis and provide us with substantial new biological insights regarding how *LY86* may be involved in candidemia susceptibility.

These results are now discussed at p10, line 238-249: *‘To test our hypothesized mechanism of action (Fig. 3D), we conducted experimental follow-up studies in THP-1 monocytes. As the candidemia risk allele is associated with reduced expression of LY86, we used siRNA knockdown of LY86 to mimic this effect. 72h after siRNA treatment, we confirmed efficient knockdown of LY86 (14.3-fold lower expression, p = 0.001) by qPCR. In line with our hypothesis, the expression of CCR2 was also reduced (33.3-fold, p = 0.0006) upon knockdown of LY86 (Fig. 3E). These 72h LY86 or control siRNA treated cells were then used in a migration assay to assess their migratory capacity towards the chemokine MCP-1 or serum-free medium as a control. After 3h incubation, we only observed migration towards MCP-1 and not the serum-free medium. Notably, the migratory capacity towards MCP-1 of the LY86 siRNA treated cells was reduced (2.0 fold, p = 0.01) as compared to control siRNA treated cells (Fig. 1F). Summarized, these results indicate that reduced expression of LY86 can reduce the migratory capacity of monocytes, potentially through reduced expression of CCR2, and thereby, may increase the susceptibility to candidemia.’*

The broader implications of our findings are now discussed at p9-10, line 228-235: *‘In addition, a previous study, in which PBMCs from 8 individuals were stimulated for 24h with microbial and fungal pathogens, showed reduced expression of LY86 after stimulation with Mycobacterium Tuberculosis(-1.20 fold, p = 1.53 x 10^-7^), Borrelia (-1.34 fold, p = 7.31 x 10^-13^), Pseudomonas Aeruginosa (-1.31 fold, p = 9.45 x 10^-9^) and Streptococcus Pseudomoniae (-1.54 fold, p = 2.01 x 10^-19^), but not Aspergillus Fumigatus (-0.012 fold, p = 0.98) [7]. Altogether, this indicates that the differential regulation of LY86 in monocytes, as seen in response to Candida, could also affect the susceptibility towards other blood-based bacterial infections.’*

**Reviewer #2: Understanding risk factors for the development of candidaemia is of high importance, however, the recent marked increase in incidence belies the fact that risk factors are predominantly iatrogenic or immunosuppression secondary to other causes. Here the authors have used a single cell sequencing analysis of candida responsive genes in tandem with previously published datasets from the authors to provide evidence supporting a role for LY86 in the response to candida.**

**Overall the study was well written and of general interest. The strengths are that the data generated are based on human samples, an agnostic approach has been taken and all data are made openly accessible to the general scientific community. I did feel that the presented results remain suggestive only however.**

By performing experimental follow-up on the generated hypothesis, we could validate our proposed mechanism of action. Therefore, the presented results now show that our integrative systems genetics approach can lead to valuable new insights in the immune response to *Candida* that can be experimentally validated.

These results are now discussed at p10, line 238-249: *‘To test our hypothesized mechanism of action (Fig. 3D), we conducted experimental follow-up studies in THP-1 monocytes. As the candidemia risk allele is associated with reduced expression of LY86, we used siRNA knockdown of LY86 to mimic this effect. 72h after siRNA treatment, we confirmed efficient knockdown of LY86 (14.3-fold lower expression, p = 0.001) by qPCR. In line with our hypothesis, the expression of CCR2 was also reduced (33.3-fold, p = 0.0006) upon knockdown of LY86 (Fig. 3E). These 72h LY86 or control siRNA treated cells were then used in a migration assay to assess their migratory capacity towards the chemokine MCP-1 or serum-free medium as a control. After 3h incubation, we only observed migration towards MCP-1 and not the serum-free medium. Notably, the migratory capacity towards MCP-1 of the LY86 siRNA treated cells was reduced (2.0 fold, p = 0.01) as compared to control siRNA treated cells (Fig. 1F). Summarized, these results indicate that reduced expression of LY86 can reduce the migratory capacity of monocytes, potentially through reduced expression of CCR2, and thereby, may increase the susceptibility to candidemia.’*

**Part II – Major Issues: Key Experiments Required for Acceptance: Please use this section to detail the key new experiments or modifications of existing experiments that should be absolutely required to validate study conclusions. Generally, there should be no more than 3 such required experiments or major modifications for a "Major Revision" recommendation. If more than 3 experiments are necessary to validate the study conclusions, then you are encouraged to recommend "Reject".**

**Reviewer #1: •	Figures in the PDF are not high enough resolution to fully evaluate—axes are blurred and difficult/impossible to read even at high magnification in adobe acrobat.**

We apologize for the blurred and difficult-to-read axes and figures. We have now increased the resolution and font size in all the figures.

**•	While their scRNAseq indicates differentially expressed genes specifically in NK cell populations, this isn’t as novel as the Abstract would make it seem. A quick pubmed reveals 241 papers on NK cells and candida. I am not a candida biologist, but a quick perusal shows lots of NK cell studies described in reviews as well (e.g. Duggan et al. 2015). The authors have not made explicit exactly what controversy their results resolve. While they cite a few papers that look at NK cells in candida infection, they perform no functional studies assessing the importance NK cells during actual infection. For example, their statement at around line 145 “Through the DE analysis we show that NK cells are strongly activated, providing extra evidence for their importance in the immune response against Candida” seems to be an overstatement of their findings. They have generated a hypothesis of NK cell importance based on the data showing NK cell transcription in response to Candida (which I don’t think is controversial), but haven’t tested NK cell importance during infection.**

The importance of NK cells in the immune response to *Candida* is less clear in literature.

Some studies state NK cells are not important in this immune response (e.g. Romani et al. 1993) whereas other studies do suggest they play an important role (e.g. Whitney et al., 2014). These opposite results may be explained by the use of different model systems or conditions under which the experiments are conducted. For example, in immunocompromised mice the depletion of NK cells increased the susceptibility to candidemia. It may be especially under these immunocompromised conditions that the role of NK cells becomes important. The risk of having blood cells directly exposed to *Candida* is increased in immunocompromised patients. Therefore, as our experimental setup directly exposes blood cells to *Candida*, this may mimic the situation that arises in these patients. Under these experimental conditions, we obtained two lines of evidence through DE and (the newly added) receptor-ligand expression analysis that support an important role of NK cells in the anti-*Candida* immune response. However, as this is not the main focus of the paper, we did not follow this up with functional experiments.

The above is discussed at p6-7, line 146-160: ‘*To follow-up on these findings, we determined whether the connectivity between each of the major cell types changed upon stimulation with Candida. For this, we calculated for each cell type their potential to interact with cells from the same or another cell type by analyzing the expression of cell type-specific receptor and ligand pairs per condition (Candida-stimulated and RPMI control), using the computational framework CellPhoneDB [16]. This analysis revealed that especially the B cells (on average 1.67-fold increase) and NK cells (on average 1.62-fold increase) gain additional potential cell-cell interactions upon stimulation with Candida (Fig. 1C). Previous studies have reached a consensus that monocytes play an important role in candidemia [17,18], but the contribution of NK cells is less clear [19,20]. Interestingly, specifically in immunocompromised mice the depletion of NK cells increased the susceptibility to candidemia [21]. As in humans, candidemia mainly affects immunocompromised patients, we hypothesize that NK cells are likely to play an important role in the human candidemia response as well. Through the DE and ligand-receptor expression analysis we show that, in addition to monocytes, also NK cells are strongly activated and are increasingly connected to other cells. This provides extra evidence for their importance in the immune response against Candida.*’

To not oversell our results, we have now changed the wording from ‘unprecedented' to ‘important’ (p1, line 25-26): ‘*This confirmed the known major role of monocytes, but more interestingly, also uncovered an important role for NK cells.’*

**•	I would have appreciated more QC for the GWAS. I couldn’t find PCA or other analysis to verify that the cases and controls are well matched. Usually, PCA is also incorporated as covariates in GWAS of unrelated individuals to control from relatedness. This should be done here or some justification provided for not doing it.**

The GWAS study has previously been published (Jaeger et al., 2019) and QC details are fully described in the supplementary data of this publication: ‘*After excluding imputed variants with a MAF < 0.10 and variants within the HLA region, we applied quality control per sample and removed 5 cases and 10 controls due to excess/reduced heterozygosity and cryptic relatedness*.’ Therefore, citing this publication suffices to inform the reader on these details.

**•	QQ plot of the complete GWAS, as well as with the individual filters in Figure 2B, would also be useful to show no inflation of test statistic and whether there was any deviation from neutral expection prior to the final filtering down to 27 response eQTLs.**

To increase the information that can be extracted from studies with relatively small sample sizes, we integrated multiple layers of information together (candidemia GWAS, bulk RNA-seq response-QTL analysis and cell type-specific scRNA-seq DE analysis). All these lines of evidence together help to prioritize the SNPs and downstream genes most important in the susceptibility to candidemia. If we were to take a random set of 27 eQTL SNPs without a response-QTL effect, we would see a similar or stronger lambda inflation value in approximately 19% of the situations (10,000 permutations). However, for the specific genetic variant that is associated with *LY86* expression (SNP rs9405943), it is the additional support that makes this specific example being interesting to follow-up as a genetic variant likely being involved in candidemia susceptibility; SNP rs9405943 is a response-QTL and its associated downstream gene *LY86* is differentially expressed in monocytes upon *Candida* stimulation. Finally, we have now also done experiments to validate that the proposed mechanism of action of this genetic variant can potentially affect candidemia susceptibility by affecting the migratory capacity of monocytes.

These results are now discussed at p10, line 238-249: *‘To test our hypothesized mechanism of action (Fig. 3D), we conducted experimental follow-up studies in THP-1 monocytes. As the candidemia risk allele is associated with reduced expression of LY86, we used siRNA knockdown of LY86 to mimic this effect. 72h after siRNA treatment, we confirmed efficient knockdown of LY86 (14.3-fold lower expression, p = 0.001) by qPCR. In line with our hypothesis, the expression of CCR2 was also reduced (33.3-fold, p = 0.0006) upon knockdown of LY86 (Fig. 3E). These 72h LY86 or control siRNA treated cells were then used in a migration assay to assess their migratory capacity towards the chemokine MCP-1 or serum-free medium as a control. After 3h incubation, we only observed migration towards MCP-1 and not the serum-free medium. Notably, the migratory capacity towards MCP-1 of the LY86 siRNA treated cells was reduced (2.0 fold, p = 0.01) as compared to control siRNA treated cells (Fig. 1F). Summarized, these results indicate that reduced expression of LY86 can reduce the migratory capacity of monocytes, potentially through reduced expression of CCR2, and thereby, may increase the susceptibility to candidemia.’*

**•	Their finding of a SNP in Ly86 as being both a response eQTL and associated with candidemia is intriguing. Though far from genome-wide significance with p~0.001, I can appreciate the value of their integrated approach in identifying this SNP. However, in the absence of stronger statistical evidence (e.g. replication of the association in another Candida GWAS), some experimental validation becomes even more crucial. Some possibilities include, demonstrating an experimental effect of reducing LY86 expression in response to Candida (and with TLR4 agonists as a control) on downstream TLR4 responses, such as CCR2 and other downstream pro-inflammatory effects, chemotaxis of cells, would seem to be highly worthwhile and quite doable. In line with that, have the authors examined whether CCR2 and other downstream targets are trans-eQTLs for their SNP. How about testing effects of altering LY86 expression specifically in monocytes or monocyte cell lines? In addition to confirming the effect of Ly86 on Candida infection, they could also consider further mapping and testing of what the causal variant is and how it affects Ly86 expression. GWAS and eQTL analysis lead to many plausible hypotheses, testing this one on Ly86 extensively would greatly elevate the significance of this paper’s findings.**

As the described GWAS study is by far the largest candidemia GWAS to date, replication in another study is currently not possible. Therefore, we combined multiple lines of evidence to support the importance of the studied genetic variants, including response-QTL analysis and DE analysis upon stimulation with *Candida*. Additionally, we have now further strengthened our story and greatly elevate the significance of our paper’s findings by performing experimental follow-up on the hypothesis that was generated by our integrative approach. By knocking down the expression of *LY86* in THP-1 monocytes using siRNA, we could mimic the effect of the candidemia-associated risk allele. Using these cells we could confirm that monocytes with lower *LY86* expression have reduced migratory capacity towards the chemokine MCP-1 as opposed to cells with normal *LY86* expression. These experiments confirmed the proposed mechanism of action of *LY86* and suggest that the lower migratory capacity of monocytes increases the risk to develop candidemia*.*

The results of this experimental follow-up are now discussed at p10, line 238-249: *‘To test our hypothesized mechanism of action (Fig. 3D), we conducted experimental follow-up studies in THP-1 monocytes. As the candidemia risk allele is associated with reduced expression of LY86, we used siRNA knockdown of LY86 to mimic this effect. 72h after siRNA treatment, we confirmed efficient knockdown of LY86 (14.3-fold lower expression, p = 0.001) by qPCR. In line with our hypothesis, the expression of CCR2 was also reduced (33.3-fold, p = 0.0006) upon knockdown of LY86 (Fig. 3E). These 72h LY86 or control siRNA treated cells were then used in a migration assay to assess their migratory capacity towards the chemokine MCP-1 or serum-free medium as a control. After 3h incubation, we only observed migration towards MCP-1 and not the serum-free medium. Notably, the migratory capacity towards MCP-1 of the LY86 siRNA treated cells was reduced (2.0-fold, p = 0.01) as compared to control siRNA treated cells (Fig. 1F). Summarized, these results indicate that reduced expression of LY86 can reduce the migratory capacity of monocytes, potentially through reduced expression of CCR2, and thereby, may increase the susceptibility to candidemia.’*

*Trans*-eQTL effects are usually much smaller than *cis*-eQTLs. Moreover, based on the scRNA-seq data the response-QTL for *LY86* seems specific to monocytes, and as a result the potential *trans* response-QTL for *CCR2* is expected to be only present in monocytes as well. Therefore, ideally to identify this potential *trans* response-QTL for *CCR2*, we would require a large scRNA-seq dataset of *Candida*-stimulated PBMCs. However, unfortunately the current scRNA-seq dataset only contains 6 individuals, which is not sufficient to identify this effect. We expect that in the near future scRNA-seq datasets will become available that have been generated in many more individuals (e.g. sc-eQTLGen consortium). These efforts will allow us to test this potential *trans* response-QTL for *CCR2* in the future.

**•	While they describe their approach as integrative, and it certainly does take different kinds of data to generate hypotheses, how well and how easily this can be generally applied is unclear. Are there more hypotheses that could be described other than Ly86?**

We followed-up the most significant candidemia-associated genetic variant. This genetic variant showed a response-QTL for *LY86* and showed reduced expression specifically in monocytes. The other candidemia-associated variants that overlapped with the 27 response-QTLs either did not show a clear cell type-specific response or the expression of the downstream gene could not be detected in the scRNA-seq data. We expect that once larger bulk or scRNA-seq datasets become available under stimulated conditions (through efforts like the eQTLGen and sc-eQTLGen consortium), it will become easier to prioritize a larger number of potentially interesting genetic variants.

**Reviewer #2: There are scant data regarding the results of the GWAS – upon consultation of the referenced paper, there is no mention of the LY86 locus in this paper for example. The authors took the summary statistics from their previous GWAS (no indication as to what P value threshold was used – but in the cited paper they stated a p Value threshold of**

All summary statistics of the GWAS study were integrated with the 27 response-QTLs that we identified using the data of *Candida*-stimulated PBMCs from a previously published bulk RNA-seq study (Li et al., 2014). So no specific p-value cut-off was used for selecting these GWAS summary statistics. In Fig. 2C we show that there is an inflation in GWAS signal for these 27 genetic variants, as opposed to what would be expected by chance.

At p8, line 196-201, we explain why we used the above strategy to prioritize downstream genes: ‘*Previously, it was shown that integrating multiple molecular datasets can help prioritize disease-relevant genes, cell types and pathways [9,10]. Therefore, as a next step, we took the GWAS summary statistics of a previously published candidemia cohort of 161 cases and 152 disease-matched controls [8] and overlaid this with our 27 response QTLs (Fig. 2B). This revealed an enrichment of candidemia-susceptibility SNPs within the Candida-response QTL SNPs (ƛ_inflation_ = 1.49) (Fig. 2C).*’

Moreover, we now specifically mention that all GWAS summary statistics were used independent of their p-value (p14-15, line 357-359): ‘*The lambda inflation was calculated by taking the GWAS p-values for each of the 27 response-QTL SNPs, regardless of whether the GWAS p-value was significant.’*

**Please use this section for editorial suggestions as well as relatively minor modifications of existing data that would enhance clarity.**

**Reviewer #1: Line 160—please list the actual n for the study being used in the text**

Whenever discussing specific results in the main text, we now added more details on the number of individuals or SNPs tested.

At p7-8, line 175-179: ‘*The rather small sample size of this study limits its predictive power, in part by the large multiple testing burden of genome-wide eQTL analysis [22]. To reduce the multiple testing burden, we limited our single nucleotide polymorphism (SNP)-gene combinations to only the 16,990 top cis SNP-gene pairs identified in the largest eQTL meta-analysis to date [23], containing unstimulated whole blood samples of 31,684 individuals.*’

At p8, line 183-185: ‘*Using this approach, a total of 1,563 and 1,637 eQTLs were found in 72 Candida-stimulated and 75 RPMI control samples, respectively (Fig. 2A, Suppl. Table 3).’*

At p8, line 189-191: ‘*This so-called response QTL analysis was performed in the 67 individuals for which both Candida-stimulated and RPMI control conditions were assessed and revealed 27 response QTLs (Suppl. Table 3).’*

**Reviewer #2: Specific points:**

**i)	What is MAST? This needs a reference (an perhaps one line describing why MAST) when first mentioned.**

MAST is the statistical method we used for DE analysis. In addition to our previous mentioning in the Materials and Methods section, we now also included additional information and a reference in the main text (p5, line 120-121): ‘*Secondly, we identified differentially expressed (DE) genes upon stimulation per cell type separately as well as in all cells together (bulk-like) by performing DE analysis with MAST [14].*’

**ii)	The authors find a common set of 41 DE genes to all cells, and these are enriched in interferon related genes. The results of other associated pathways are not provided and the methods used for pathway analysis are not specified (which pathways? GOBP/ KEGG? Which algorithms? Which tests?). This needs to rectified in a revision – if pathway analysis is performed then all results should be provided (even as supplementary) and methods used.**

The REACTOME pathways are used as an input for the pathway analysis using the ToppFun package. We have now included additional details on this procedure in the Materials and Methods section (p13, line 311-314): *‘Genes that were differentially expressed in all cell types (i.e. core genes) and each cell type individually were used as input for the ToppFun functional enrichment analysis using the REACTOME pathway [41]. P-values were calculated using the probability density function and were Bonferroni corrected.’*

Additionally, we have now included Supplementary Table 2 in which the top10 most significantly enriched REACTOME pathways are depicted for the DE genes of each cell type separately, for all cells combined (bulk-like sample) and for the 41 DE core genes that were identified in each cell type.

**Analysis of Supplementary Table 1 demonstrates that the top common genes are dominated by ISG/IFI/OAS members. This is an interesting finding, but these genes are typically induced by Type 1 interferons. Type 1 interferons elicit a profound effect across multiple cell types and the methodology employed prevents determination of genes differentially expressed secondary to candida as opposed to those that are differentially expressed secondary to cytokines released from another cell type (e.g. interferon released from innately activated monocytes/ plasmacytoid dendritic cells). NK cells are particularly sensitive to type 1 interferons and this might explain the marked differential response in this (prolonged) incubation. To truly demonstrate NK cells are directly responsive to candida the experiment would need to be performed without other cell types present. The authors wish to discuss this in their conclusions.**

We now mention that our experimental set-up does not allow us to distinguish between direct and indirect responses upon *Candida* stimulation at p6, line 131-133: ‘*However, please note that during this prolonged incubation of 24h, it is not possible to distinguish between direct and indirect responses upon Candida stimulation.’*

**iii)	The authors propose a model whereby the LY86 risk allele leads to increased downregulation of the gene upon exposure to candida, resultant in increased TLR4 signalling and increased downregulation of CCR2. This would suggest that in monocytes, independently of genotype, the post exposure expression of LY86 is anti-correlated with CCR2 – is this the case? Moreover, the model would suggest increased TLR4 signalling too – are there detectable trans effects of this SNP commensurate with this model?**

We are sorry there is some confusion about the proposed model. We propose that the *LY86* risk allele leads to increased down regulation of the gene upon exposure to *Candida*. Thereby, the *LY86*-mediated inhibition of TLR4 signaling in monocytes is suppressed. This higher activity of TLR4 results in increased suppression of *CCR2* (so lower *CCR2* expression levels). In line with this proposed model, we show in Fig. 1C that this positive correlation between *LY86* and *CCR2* expression exists in our scRNA-seq data (both before and after *Candida* stimulation).

*Trans*-eQTL effects are usually much smaller than *cis*-eQTLs. Moreover, based on the scRNA-seq data the response-QTL for *LY86* seems specific to monocytes, and as a result the potential *trans* response-QTL for genes downstream of TLR4 signaling (e.g. *CCR2*) is expected to be only present in monocytes as well. Therefore, ideally to identify these potential *trans* response-QTLs downstream of TLR4 signaling, we would require a large scRNA-seq dataset of *Candida*-stimulated PBMCs. However, unfortunately the current scRNA-seq dataset only contains 6 individuals, which is not sufficient to identify this effect. We expect that in the near future scRNA-seq datasets will become available that have been generated in many more individuals (e.g. sc-eQTLGen consortium). These efforts will allow us to test these potential *trans* response-QTLs in the future.

**iv)	For the authors’ model to stand it would require the response QTL to form pQTL. Given the weak statistical significance supporting the association with LY86 I think this model is currently rather speculative and would be greatly strengthened with more evidence to support LY86 in the response to candida. For example, does modulating the expression of LY86 alter the migratory capacity of monocytes to MCP-1? Some form of orthogonal experiments would be beneficial in supporting the currently speculative conclusions.**

As suggested, we have now included an experiment in which we test the migratory capacity of THP-1 monocytes to MCP-1 after 72h knock down of *LY86* expression using siRNA. This experiment provides substantially more evidence supporting our hypothesis regarding the mechanism through which the SNP rs9405943 impacts candidemia susceptibility.

We have added the following sections to the text to present these results (p10, line 238-249): *‘To test our hypothesized mechanism of action (Fig. 3D), we conducted experimental follow-up studies in THP-1 monocytes. As the candidemia risk allele is associated with reduced expression of LY86, we used siRNA knockdown of LY86 to mimic this effect. 72h after siRNA treatment, we confirmed efficient knockdown of LY86 (14.3-fold lower expression, p = 0.001) by qPCR. In line with our hypothesis, the expression of CCR2 was also reduced (33.3-fold, p = 0.0006) upon knockdown of LY86 (Fig. 3E). These 72h LY86 or control siRNA treated cells were then used in a migration assay to assess their migratory capacity towards the chemokine MCP-1 or serum-free medium as a control. After 3h incubation, we only observed migration towards MCP-1 and not the serum-free medium. Notably, the migratory capacity towards MCP-1 of the LY86 siRNA treated cells was reduced (2.0-fold, p = 0.01) as compared to control siRNA treated cells (Fig. 1F). Summarized, these results indicate that reduced expression of LY86 can reduce the migratory capacity of monocytes, potentially through reduced expression of CCR2, and thereby, may increase the susceptibility to candidemia.’*

**Figures:**

**Figure 1B – what is being depicted here? “number of unique cell types where a specific gene has been found to be differentially regulated” – if number &gt;1 then not unique. Relabel as “number of cell types gene differentially regulated in” or similar. The bottom of each bar has a black line, yet this is not contiguous with the x axis (which does not exist). Either place an axis line or remove this darkline as I thought that this must represent something at first.**

For each gene we determined in how many cell types the gene is differentially expressed, and this is depicted in figure 1B. We have followed the reviewer's suggestion and changed the labelling of the x-axis to ‘*Number of cell types a gene is differentially regulated in*’. Moreover, we have removed the black bar at the bottom of each of the bars to prevent any confusion.

**The labelling on Figure 1C is confusing - it would be better for the label of interferon regulated genes to be to the right of the alternating dark and light grey indicators above the labels for cell type. As it stands, until the legend has been consulted, it is unclear whether it is labelling entire heatmap.**

To prevent confusion, we have changed the figure in the way the reviewer suggested by moving the labelling of the interferon genes to the side of this figure.
