# Supplementary material for: Integrating GWAS with bulk and single-cell RNA-sequencing reveals a role for LY86 in the anti-Candida host response
Source: PLoS Pathog. 2020 Apr 6;16(4):e1008408. doi: 10.1371/journal.ppat.1008408 (PMC7173933; doi:10.1371/journal.ppat.1008408)
Supplement: Attachment — Submitted filename: Reviewer Comments.docx [file ppat.1008408.s011.docx]

**Reviewer Comments**

We appreciate that both reviewers have taken the time to have another careful look at our manuscript. The reviewers mention that their comments have now been addressed in full and that our study is of general interest to the field and warrants publication in its present form without further revisions. They only point to some extra clarification of how the experimental work (Fig. 3E, 3F) is related to the *Candida* data (from Fig. 1-3C) and extra clarification of the proposed working mechanism as depicted in Figure 3. We have now further clarified this Figure to better represent the mechanism as discussed in the main text. We have provided a point-point reply to each of the questions that have been raised. Reviewer comments are in **Verdana Bold**, our responses are in plain Verdana, and adjustments to the text in *cursive* *Verdana*.

**Part I- Summary**
**Reviewer #1: (No Response)
Reviewer #2: The authors have addressed my comments in full and their study is of general interest to the field and warrants publication in its present form without further revisions.

Part II – Major Issues: Key Experiments Required for Acceptance**
**Reviewer #1: In addressing my previous major concerns, the authors have softened claims about the novelty of the importance of NK cells in candidemia while also providing more evidence for this and have now included promising data validating their claims regarding LY86. In general, the additions and edits to the manuscripts have substantially improved the paper. Most importantly, they show that knockdown of LY86 in THP-1s results in decreased CCR2 expression, causing reduced chemotaxis in response to CCL2 (MCP-1). While these experiments appear carefully done and knockdown and phenotype are robust, they should be connected more to the Candida results in the rest of the paper. Ideally, this would be done by showing migration is decreased in monocytes following stimulation with Candida, and that migration is even lower in AA individuals vs. GG individuals. If such cells are not available for that, I think that at least showing that Candida inhibits migration of THP1s to CCL2 (or citing a reference that does demonstrate that) would be valuable in providing further evidence that your model is correct, possibly revealing a mechanism of Candida immune suppression, and connecting it more to the Candida work in the rest of the paper.**

The experimental follow-up is connected to *Candida* susceptibility in the following manner: 1. Identification of 27 genetic variants that modulate downstream expression in a Candida-responsive manner (see section ‘Identification of *Candida*-response QTLs using bulk RNA sequencing’); 2. Overlay these genetic variants with the results of a candidemia GWAS, which identifies that the genotype that results in a higher risk of developing *Candida* infection (at SNP rs2103635) is in near perfect LD with the genotype (the candidemia-risk allele *A* at rs9405943) that is associated with a stronger downregulation of *LY86* upon *Candida* stimulation (see section ‘Prioritization of *LY86* as a potential key driver gene for candidemia’); 3. The scRNA-seq data that shows that the downstream effect of this candidemia-risk allele upon *Candida* stimulation is taking place in the monocytes (see section ‘Prioritization of *LY86* as a potential key driver gene for candidemia’); 4. The experimental follow-up of downregulating *LY86* using siRNA in THP-1 cells mimics monocytes that are being stimulated with *Candida* while having a genotype that is associated with having the candidemia-risk allele at rs9405943 that is associated with an increased risk of developing *Candida* infection (and resulting in stronger reduction of LY86 expression upon *Candida* stimulation) (see section ‘Functional validation of the role of LY86 in monocytes’). Therefore, the findings that can be extracted from this experimental follow-up, are directly related to *Candida* infection.

We now specifically mention how the experimental work is directly related to the *Candida* work in the rest of the paper, including references to the relevant figures (p10, line 241-243): *‘As the candidemia risk allele in combination with Candida stimulation is associated with reduced expression of LY86 specifically in the monocytes (Fig. 2C, 3A, 3C), we used siRNA knockdown of LY86 to mimic this effect.’*

**Additionally, Tobias Hohl’s lab seems to have provided evidence that your mechanism could be very important in vivo based on Ngo et al 2014, which you cite earlier in your paper, but not for the finding that CCR2-dependent chemotaxis of monocytes is crucial in mouse candidiasis. Again, I think the new RNAi expt is a good addition to the paper. But showing the inhibition of migration by Candida and citing how crucial CCR2+ monocytes are in systemic candidiasis could elevate the importance of your work further.**

Thank you for this suggestion. We agree that citing the Ngo et al. 2014 paper further supports the importance of CCR2-mediated monocyte trafficking in the anti-*Candida* response. Therefore, we now discuss this paper while discussing the proposed mechanism of action of the role of LY86 in the anti-*Candida* response (p9, line 226-228): ‘*In line with this, in mice the trafficking of CCR2-dependent inflammatory monocytes has been shown to play an essential role in fungal clearance during systemic candidiasis in the first 48h of infection [18].*’

**One other point regarding Figure 3D: The model does not match what is described in the Results. This figure shows a competitive model between LY86 and LY96 for binding of TLR4. It would be helpful to indicate LY86 is also known as MD-1 (and LY96 (MD-2)). More importantly, based on the Results text and a quick perusal of the literature, it would seem that a more likely model involves RP105, which appears to be a divergent TLR with most sequence similarity to TLR4. A model depicting decreased RP105/MD-1 complex signaling being downregulated by Candida, and to a greater extent with the A allele, to decrease CCR2 levels and chemotaxis would seem to be what is supported by their data and the literature.**

We agree that Figure 3 represented a simplification of the proposed working mechanism, and as a result might have implicated another mechanism as what has been described in the main text. Therefore, we have now adapted Figure 3 to better represent the actual working mechanism by including the RP105-Ly86 complex and explaining that this complex directly binds and inhibits the TLR4-Ly96 mediated CCR2 repression (and thereby the monocyte recruitment towards MCP-1). Additionally, we have adapted the accompanying figure legend (p23, line 623-628): *‘LY86 (aka MD-1) can form a complex with RP105 (aka CD180) and this complex directly binds to the LY96(aka MD-2)-TLR4 complex, thereby inhibiting TLR4 signaling. However, in individuals with the rs9405943 candidemia-risk allele, LY86-RP105 complex formation is reduced, and as a result, LY96-TLR4 signaling is increased. As a consequence, TLR4-mediated chemokine receptor 2 (CCR2) repression increases, which reduces monocyte recruitment towards MCP-1 (aka CCL2) and increases candidemia susceptibility.’*

**Reviewer #2: NA**

**Part III – Minor Issues: Editorial and Data Presentation Modifications**
**Reviewer #1: (No Response)
Reviewer #2: NA**

Figure Files:

While revising your submission, please upload your figure files to the Preflight Analysis and Conversion Engine (PACE) digital diagnostic tool, [https://pacev2.apexcovantage.com](https://eur03.safelinks.protection.outlook.com/?url=https%3A%2F%2Fpacev2.apexcovantage.com%2F&data=02%7C01%7Cm.g.p.van.der.wijst%40umcg.nl%7Cc4798c3760674bc64a6f08d7abea132c%7C335122f9d4f44d67a2fccd6dc20dde70%7C0%7C0%7C637166894140048841&sdata=etYpv5IM44w5%2BJlRA4UUNnhyhfPfQYyvaTUjtF7k9V0%3D&reserved=0). PACE helps ensure that figures meet PLOS requirements. To use PACE, you must first register as a user. Then, login and navigate to the UPLOAD tab, where you will find detailed instructions on how to use the tool. If you encounter any issues or have any questions when using PACE, please email us at [figures@plos.org](mailto:figures@plos.org).

Data Requirements:

Please note that, as a condition of publication, PLOS' data policy requires that you make available all data used to draw the conclusions outlined in your manuscript. Data must be deposited in an appropriate repository, included within the body of the manuscript, or uploaded as supporting information. This includes all numerical values that were used to generate graphs, histograms etc.. For an example see here: <http://www.plosbiology.org/article/info%3Adoi%2F10.1371%2Fjournal.pbio.1001908#s5>.

Reproducibility:

To enhance the reproducibility of your results, PLOS recommends that you deposit laboratory protocols in protocols.io, where a protocol can be assigned its own identifier (DOI) such that it can be cited independently in the future. For instructions see [http://journals.plos.org/plospathogens/s/submission-guidelines#loc-materials-and-methods](https://eur03.safelinks.protection.outlook.com/?url=http%3A%2F%2Fjournals.plos.org%2Fplospathogens%2Fs%2Fsubmission-guidelines&data=02%7C01%7Cm.g.p.van.der.wijst%40umcg.nl%7Cc4798c3760674bc64a6f08d7abea132c%7C335122f9d4f44d67a2fccd6dc20dde70%7C0%7C0%7C637166894140048841&sdata=epiod4bNBxOUbZ1ZDr5dKLfuVZsyUD0VlsflGQY5RTU%3D&reserved=0)

*In compliance with data protection regulations, you may request that we remove your personal registration details at any time.* [*(Remove my information/details)*](https://eur03.safelinks.protection.outlook.com/?url=https%3A%2F%2Fwww.editorialmanager.com%2Fppathogens%2Flogin.asp%3Fa%3Dr&data=02%7C01%7Cm.g.p.van.der.wijst%40umcg.nl%7Cc4798c3760674bc64a6f08d7abea132c%7C335122f9d4f44d67a2fccd6dc20dde70%7C0%7C0%7C637166894140058844&sdata=7egnniian6P1Gtof5SwhyuK5WymFu9s2s9jI4bcvKog%3D&reserved=0)*. Please contact the publication office if you have any questions.*
